# The Rare Case of a Sertoli Cell Tumor of the Adrenal Gland in a Man With Pathological Dexamethasone Suppression Test: A Case Report

**DOI:** 10.7759/cureus.115277

**Published:** 2026-08-27

**Authors:** Andreas Schroll, Julia Mühlhäusser, Martin Bolli, Wilhelm Nimphius, Joern-Markus Gass

**Affiliations:** 1 General and Visceral Surgery, Cantonal Hospital of Lucerne, Lucerne, CHE; 2 Pathology, Cantonal Hospital of Lucerne, Lucerne, CHE

**Keywords:** adrenal disease, adrenal gland neoplasms, adrenal tumours, leydig and sertoli cells, sertoli cell tumor, sex cord stromal tumors, sex cord tumor

## Abstract

Sertoli cell tumors are rare gonadal stromal tumors. Extragenital manifestations, particularly in the adrenal gland, are exceptionally uncommon. We present a case of an adrenal Sertoli cell tumor in a male patient, presenting with a pathological (1 mg overnight) dexamethasone suppression test.

An 82-year-old otherwise healthy man was referred for evaluation of an incidentally detected left adrenal mass. The patient was asymptomatic with no clinical signs of catecholamine excess, hypercortisolism, hyperaldosteronism, or B-symptoms. Initial CT scan revealed a 44 × 42 mm lesion, which increased to 50 × 46 mm within three months and showed suspicious washout characteristics. Endocrinological work-up showed a pathological (1 mg overnight) dexamethasone suppression test, while metanephrines and aldosterone testing were normal. Tumor markers [alpha-fetoprotein (AFP), beta-human chorionic gonadotropin (β-HCG)] were unremarkable. A hormonally active adrenal adenoma with autonomous cortisol production was suspected. After discussion at the interdisciplinary tumor board, robotic-assisted adrenalectomy was performed. Complete resection was achieved without complications; the patient was discharged on the third postoperative day. Histopathological examination showed a Sertoli cell tumor with complete excision and no evidence of local infiltration or malignancy. Immunohistochemistry showed positivity for cluster of differentiation (CD)56, CD99, synaptophysin, and partial positivity for SF1, calretinin, and inhibin. Chromogranin A and MART1 were negative. Molecular analysis revealed no FOXL2 or DICER1 mutations. Postoperatively, hydrocortisone replacement was initiated due to a submaximal cortisol response to adrenocorticotropic hormone (ACTH) stimulation testing. At six-month follow-up, CT imaging showed no recurrence or metastases, and tumor markers remained normal.

Adrenal Sertoli cell tumors represent an extremely rare entity lacking specific management guidelines. In this case, robotic-assisted adrenalectomy proved safe and feasible. We observed good short-term oncological and clinical outcomes. Further reports are needed to clarify hormonal associations and establish standardized follow-up and treatment strategies.

## Introduction

Sertoli cell tumors are rare diseases that arise from the gonadal sex cords and are classified as testicular sex cord stromal tumors [1]. Testicular sex cord stromal tumors account for approximately 3-5% of all testicular tumors, of which 1% can be attributed to Sertoli cell tumors [1,2]. Sertoli cell tumors can be further subdivided into general, large-cell calcifying, intratubular hyalinizing, and sclerosing cell tumors [3,4]. According to the literature, approximately 10-25% of these tumors are considered malignant [4,5]. The incidence of metastases is estimated at 10% [1], with retroperitoneal lymph nodes, lungs, bones, and inguinal lymph nodes being the most common sites of metastases [2].

In most cases, Sertoli cell tumors do not cause any symptoms and are therefore primarily asymptomatic [1]. Extragenital localization of Sertoli cell tumors is remarkably rare. To date, there has only been one reported case in the literature of a Sertoli cell tumor located in the adrenal gland [5].

In this paper, we present the second case of a Sertoli cell tumor in the adrenal gland, to the best of our knowledge. This case report was presented as a visual abstract at the 2026 Annual Meeting of the Swiss College of Surgeons on 11-12 June 2026.

## Case presentation

An 82-year-old patient in good general health attended a medical consultation for further evaluation of an incidentally detected mass in the left adrenal gland. The patient was completely asymptomatic regarding the tumor. There was no flank or abdominal pain, only occasional nonspecific headache and leg pain. The patient reported a planned weight loss from 92kg to 74kg over the last three years with dietary adjustments and increased physical activity. Notably, there were no symptoms of increased catecholamine release, hypercortisolism, or B-symptoms.

The patient's comorbidities included coronary heart disease with concomitant atrial fibrillation, arterial hypertension, and a previous stent placement. Furthermore, the patient had dyslipidemia and osteoporosis. No personal history of carcinoma was reported. 

**Figure 1:**
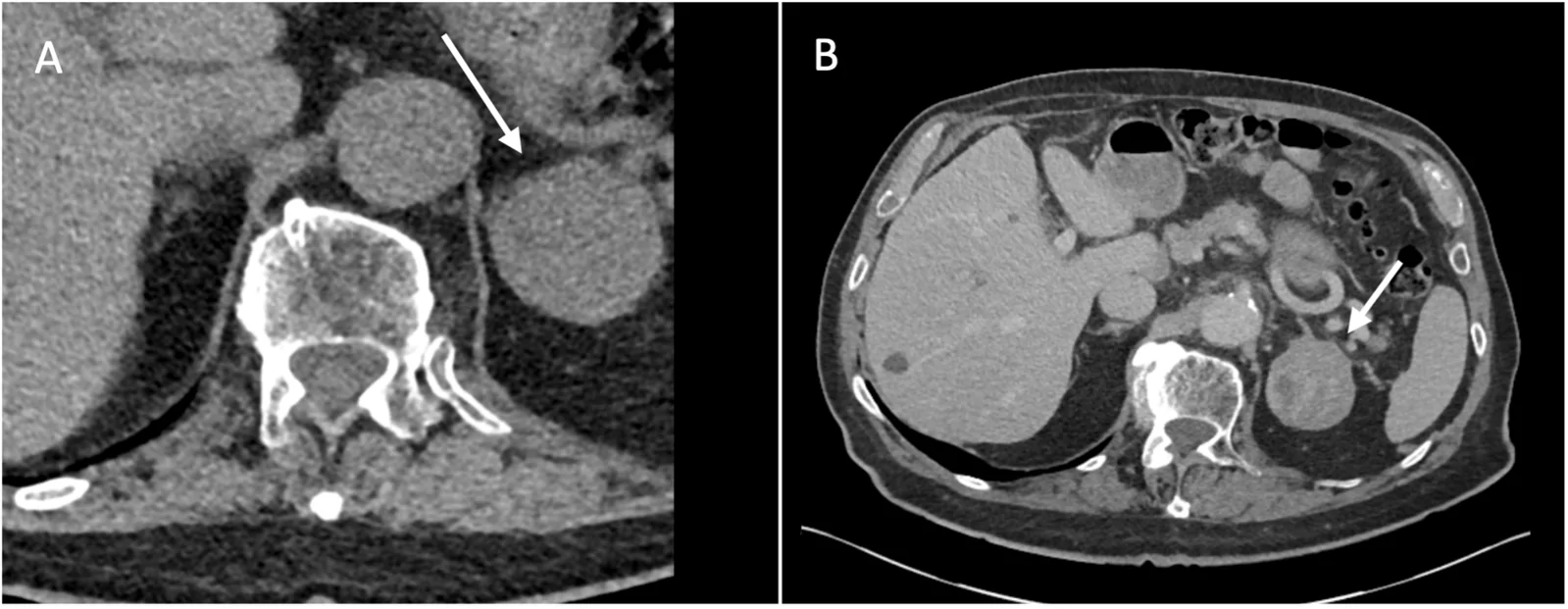
Tumor progression of the adrenal mass in three months (approx. 6mm). (A) Initial CT scan of the lumbar spine, (B) Follow-up CT scan of the abdomen (absolute washout 45%).

The adrenal mass was initially discovered as an incidental finding during a CT scan (44 x 42 mm) for back pain (Figure 1A). In a follow-up abdominal CT scan after three months, the tumor had grown in size (50 x 46 mm) and showed wash-out characteristics suspicious for carcinoma (absolute wash-out 45%) (Figure 1B).

**Figure 2:**
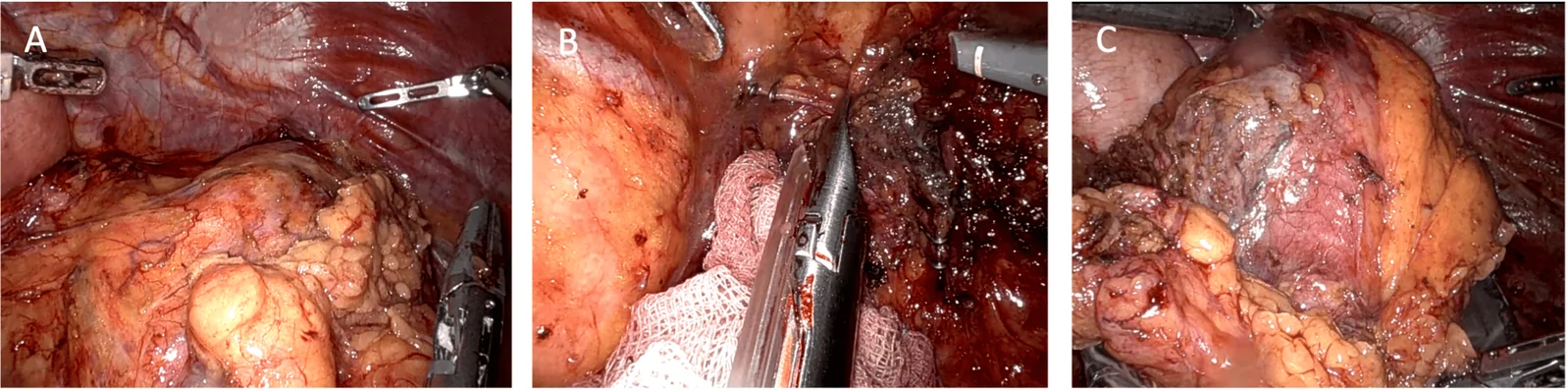
Intraoperative view during robotic-assisted adrenalectomy. (A) Visualization of the tumor prior to preparation, (B) Clamping of tumor-supplying vessels,(C) Removal of the tumor after complete resection.

Additionally, the patient showed pathological results in a (1 mg overnight) dexamethasone suppression test. The patient was therefore assessed by the department of endocrinology. Given the non-suppressible cortisol value, autonomous cortisol secretion by the tumor had to be assumed. There was no evidence of aldosterone co-secretion in the context of hyperaldosteronism. A metanephrine test showed no abnormal findings (0.42 nmol/l), making the presence of a pheochromocytoma unlikely. In addition, alpha-fetoprotein (4.29μg/L) and beta-HCG (<0.6 IU/L) presented normal results. After discussion at the interdisciplinary tumor board, robotic-assisted left adrenalectomy was the next step. During the operation, a mass measuring approximately 6 cm was found in the left retroperitoneum (Figure 2A). Due to kinking of the aorta, the left renal and adrenal veins could not be visualized, necessitating sequential clamping of the tumor-supplying vessels (Figure 2B). This resulted in the decision to remove the adrenal gland retrograde (Figure 2C).

**Figure 3:**
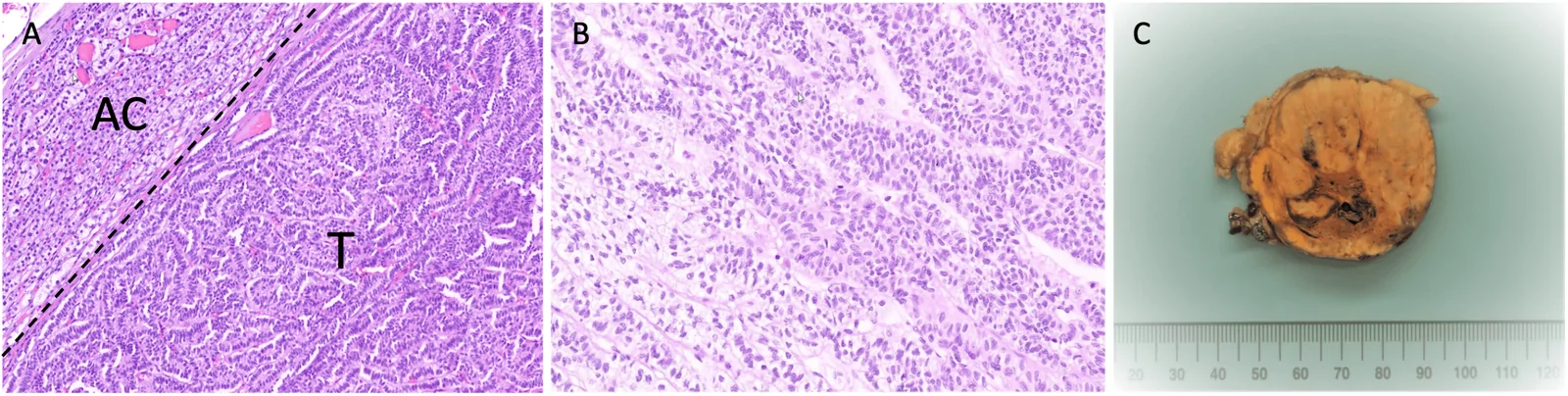
Microscopic visualization of the resected adrenal specimen (hematoxylin and eosin stain). (A) Magnification x40, representation of tumor cells (T) demarcated from the intact peripheral adrenal cortex (AC) by a dashed line. (B) Magnification x100, close-up view of the Sertoli cells arranged in a characteristic tubular growth pattern. (C) Cross-sectional image of the specimen.

The tumor was completely resected during surgery and sent for histological analysis. The analysis showed a well-circumscribed, benign nodular lesion with a yellowish cut surface, consistent with a Sertoli cell tumor. Complete excision was confirmed, with no evidence of local infiltration or malignancy (Figure 3). 

Immunohistochemical analysis of the tumor revealed positivity for CD56, CD99, synaptophysin, and partial positivity for SF1, calretinin, and inhibin. Chromogranin A and MART1/Tyros proved negative. Furthermore, molecular analysis of the tumor showed no evidence of FOXL2 and DICER1 gene mutations.

**Figure 4:**
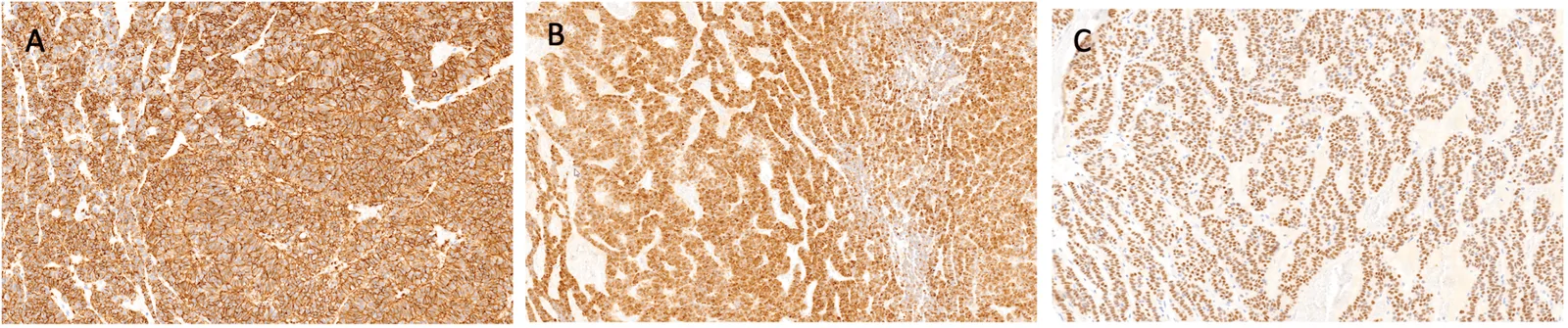
Visualization of immunohistochemistry, magnification x100. (A) Strong membranous positivity for CD56. (B) Positive staining for inhibin in the tumor cells. (C) Nuclear positivity for SF1, consistent with sex cord-stromal differentiation.

The patient was discharged from the hospital on the third postoperative day; no complications occurred. Hydrocortisone substitution therapy was initiated following consultation with the endocrinology department during the hospitalization. Endocrinological follow-up two months after surgery revealed a submaximal increase in the adrenocorticotropic hormone (ACTH) stimulation test, necessitating the continuation of hydrocortisone therapy. Alpha-fetoprotein (5.24 μg/L) and beta-HCG (<0.6 IU/L) continued to show normal parameters. Six months postoperatively, a CT scan of the abdomen showed no evidence of tumor recurrence or metastasis.

## Discussion

This case report illustrates the very rare case of an adrenal incidentaloma, which was discovered during a CT scan due to back pain. Only one case of a Sertoli cell tumor in the adrenal gland has been described to date [5], and the optimal treatment remains to be discussed, as there are no specific guidelines on this subject yet. 

In cases of adrenal incidentaloma and diagnostic workup, a preoperative histological examination by means of a tumor biopsy is usually not recommended. A biopsy may result in the spread of adrenal cancer or severe hemodynamic complications in cases of pheochromocytoma or paraganglioma. Furthermore, adrenal biopsies are becoming less and less important due to constantly improving imaging techniques. Therefore, the necessity of a biopsy should be discussed in a multidisciplinary setting and only performed if the tumor is not distinctly benign based on imaging alone and the biopsy has therapeutic relevance for the patient [6]. 

As a treatment method, surgical resection is recommended for unilateral adrenal lesions with radiological suspicion of malignancy in the absence of metastases, as well as lesions with relevant hormone excess [6,7]. As our patient had a possible carcinoma and, in addition, suspected autonomous cortisol secretion due to the non-suppressible cortisol level in the (1 mg overnight) dexamethasone suppression test, the subsequent procedure was discussed and planned in an interdisciplinary setting. In our case, following initial diagnosis, a robotic-assisted adrenalectomy was performed with complete resection of the tumor. A short hospitalization was required due to the minimally invasive approach. Following histological examination, the case was discussed at the interdisciplinary tumor board, which recommended follow-up with CT imaging and tumor marker assessment [beta-human chorionic gonadotropin (β-HCG), alpha-fetoprotein (AFP)] six months after the operation. 

No adjuvant therapy was administered to our patient postoperatively, as experience and evidence in the literature are limited, without clear recommendations for adjuvant treatment in the case of a Sertoli cell tumor [2]. Consequently, adjuvant treatment should be adapted individually based on risk factors and the clinical context of the patient.

In the only other published case report of an adrenal Sertoli cell tumor, Ivanis et al. observed that their patient did not require antihypertensive therapy postoperatively due to normotension. This led them to consider a possible correlation between arterial hypertension and the metabolic activity of Sertoli cell tumors. In addition, two cases of female patients with renin-producing Sertoli cell tumors associated with arterial hypertension have been described in the literature [5]. Our patient was likewise diagnosed with arterial hypertension before his initial presentation at our center. However, antihypertensive therapy was continued postoperatively without adjustment due to persistent hypertension. During our diagnostic workup, aldosterone co-secretion in the context of hyperaldosteronism could be excluded.

Furthermore, our patient showed pathological results in the preoperative (1 mg overnight) dexamethasone suppression test, indicating autonomous cortisol secretion. A postoperative ACTH stimulation test demonstrated only a submaximal increase in cortisol levels, necessitating hydrocortisone substitution therapy. This finding might suggest a potential association between autonomous cortisol secretion and Sertoli cell tumors. However, Ivanis et al. reported normal cortisol and ACTH levels as well as a normal (1 mg overnight) dexamethasone suppression test [5]. Isolated reports in the literature describe adrenocortical hyperplasia in large cell calcifying type Sertoli cell tumors, which may provide a possible explanation for our observation [4]. Further case reports are warranted to elucidate this association in greater detail.

Regarding immunohistochemistry, Ivanis et al. similarly observed a positive result for SF1 and CD56, along with negative staining for chromogranin A [5]. Furthermore, concerning molecular analysis, the literature indicates that DICER1 variants are commonly observed in both ovarian and testicular Sertoli cell tumors [8,9]. In ovarian Sertoli-Leydig-Cell-Tumors, DICER1 mutations are associated with moderate to poor differentiation and a worse prognosis. The presence of a FOXL2 mutation can help to distinguish between stromal tumors and gonadal tumors [8]. 

We consider the short duration of follow-up without knowledge of the further outcome, regarding possible tumor recurrence and cortisol levels, to be a limitation of our case. Furthermore, the follow-up of the patient's arterial hypertension would be of interest.

## Conclusions

Treatment of Sertoli tumors of the adrenal gland is particularly challenging, as it is a very rare entity and guidelines are lacking. Further research is needed to establish specific guidelines. Based on our experience in this case report, robotic-assisted surgical resection of this tumor is a safe and feasible treatment approach.
